# Migration, language and secondary trauma: The hidden challenges for mental health researchers

**DOI:** 10.1371/journal.pmen.0000584

**Published:** 2026-04-08

**Authors:** Rowan Madzamba, Asithandile Nozewu, Warona Mateane, Kitso Setswe, Christine Anthonissen, Leslie Swartz, Mike Mösko

**Affiliations:** 1 Psychology Department, Stellenbosch University, Stellenbosch, South Africa; 2 Department of Psychiatry, Stellenbosch University, Faculty of Medicine & Health Sciences, Cape Town, South Africa; 3 Department of General Linguistics, Stellenbosch University, Stellenbosch, South Africa; 4 Department of Medical Psychology, University Medical Center Hamburg-Eppendorf, Hamburg, Germany; 5 Magdeburg-Stendal University of Applied Sciences, Stendal, Germany; PLOS: Public Library of Science, UNITED KINGDOM OF GREAT BRITAIN AND NORTHERN IRELAND

While existing literature on secondary traumatization has largely focused on family members and mental health practitioners, few studies have considered its impact on researchers themselves [1–11]. Between October 2023 and April 2024, we interviewed 71 participants, including traumatized immigrants, as part of an international study on multilingualism and migrant mental health care in Germany, Romania, China, the Netherlands, and South Africa (MiM2M). Drawing on our experiences interviewing traumatized immigrants in South Africa, here we present a reflexive analysis, opinions, and recommendations aimed at shaping expectations and guiding researchers in preparing for studies involving traumatized migrant populations. To contextualize our perspectives and recommendations, we outline the interviewers’ backgrounds under pseudonyms: Serbal (33), a Zimbabwean migrant; Nyasha (32) and Chiedza (29), South African internal migrants. None are trained mental health practitioners or counsellors.

## First day experience and its aftereffects

The first day of conducting what we came to regard as “trauma research” was the most challenging. Although we had an interview guide developed with expert input and extensively piloted, this initial day set the tone, shaping our expectations and approach to questioning participants throughout the data-gathering process. We had read extensively on migration, language and trauma, and had our own lived experience in this regard as well as senior mental health professional and academic backgrounds. Nevertheless, our experience on the first day of interviews left us feeling emotionally unprepared for the stories shared by participants.

In our preparations, we had anticipated that by opening the discussion with a general question about participants’ migration histories, we would convey our personal interest and set participants at ease, thereby creating a psychologically safe setting. However, the first participant responses to what we had framed as neutral questions changed everything. The first migrant participants narrated stories of the trauma they had experienced on their journeys, and difficulties integrating into their host community context. Their experiences left us deeply shaken. The second day was similar in terms of participant narratives, but the first day’s experience had assisted in shaping our expectations, slightly better preparing us to receive the experiences related to the participants.

As we progressed through the days of scheduled interviews, we became somewhat accustomed to the stories, and every narrative carried details that impacted on us emotionally. As interviewers, our initial experience of being profoundly impacted on by each new account progressed to an experience of increased numbing. We battled with the conflict of being empathetic listeners and fulfilling the assignment of completing the interview guide in the space of approximately an hour. Thus, we had progressed from our raw responses on the first day, to the goal-directed completion of the data collection among migrant participants in the days that followed. We had become more focused on steering the interviews towards those aspects which would directly inform the larger study, while also protecting ourselves from being drawn into conversations on topics which we were not fully equipped to manage. We had to distance ourselves from participants emotionally and relationally, possibly at the cost of the support they expected. This expectation persisted despite our clear verbal and written explanations of the project’s aims before the interviews.

After completing the interviews, we realised we could not have been fully prepared, as each story carried a distinct emotional impact. Many participants cried while recounting their experiences, and we offered tissues as a gesture of support. While most appreciated this, one participant perceived it as an expectation of his tears, recalling ineffective past therapy. We learned that even well-intended gestures can be interpreted differently depending on personal histories.

## Meeting (or not meeting) expectations: The politics of helplessness

One of the first challenges was migrants’ expectations for help beyond the study, such as jobs, food parcels, documentation, and medical bills. This created an ongoing ethical and emotional dilemma: we could listen but not offer tangible, long-term support. We learned that ethical engagement required balancing empathy with professional boundaries, building trust while being clear about our roles and limits. Although we were not therapists, some interviews felt therapeutic, with migrants expressing gratitude for being heard.

This emotional tension was further complicated by our reliance on informal, untrained interpreters. Watching the interpreter and participant converse in their language, we could see the intensity of participants’ traumatic narratives unfold. Even without understanding every word, the affective weight was palpable. Such exposure to others’ emotional turmoil left us feeling drained and overwhelmed.

Importantly, these encounters did not affect us uniformly. Serbal, drawing on his inter-country migration background and similar past experiences, related closely to participants and appeared more resilient. In contrast, the two South African researchers, new to sustained engagement with migrant precarity, were more visibly affected. This difference illustrates how prior exposure shapes the emotional impact of such research. For the South African researchers, hearing firsthand migrant stories profoundly reshaped their perspectives on migrants in South Africa.

## Discussion

A key feature of our experience as a team of interviewers was that, despite considerable preparation prior to fieldwork, we were confronted with histories unfamiliar to us and narratives that were emotionally and intellectually challenging to process. Over time, the emotional toll became evident: we felt both numbed and deeply affected. Our experience resembled secondary traumatization, compounded by language barriers that introduced additional layers of complexity. Although our assignment was to conduct research, participants often perceived us as potential advocates or sources of practical assistance. When we were unable to meet these expectations, a gap emerged between research objectives and participants’ lived realities. This therefore marks that in conducting research with traumatized migrants, we must expect and prepare for moments of feeling helpless while listening to migrants’ stories.

This experience highlights the need for clear informed consent and transparency about the study’s limits. Clearly stating what the research can and cannot offer protects both researchers and participants. Involving individuals with lived experience in study design and consent processes can further align expectations and reduce risk. Our experience emphasizes the importance of training researchers to manage traumatic narratives of vulnerable participants, and the necessity of access to mental health support and regular debriefing sessions before and after interviews have been completed [12]. While participants generally reported relief in sharing their stories, this does not absolve researchers of ethical responsibility. The emotional strain we experienced echoes what trauma literature describes as “moral injury” [13]. We have therefore provided some recommendations that are aimed at ensuring the psychological safety of researchers as well as study participants (Table 1).

**Table 1:** Recommendations.

Level of Responsibility	Recommendation	Rationale (Drawn from Our Experience)
**Principal Investigators**	Provide mandatory psychological preparedness training before fieldwork and ensure clear informed consent outlining the study’s limits, developed with lived experience input.	Early interviews were destabilising despite preparation; clear expectation management protects both researchers and participants.
	Incorporate structured debriefing, mental health support, and service partnerships into trauma-focused research design.	Supports researcher wellbeing and ethical responsiveness beyond data collection.
**Research Teams**	Prepare interviewers for strong emotional reactions during early interviews and normalise shock, distress, and later emotional numbing.	Initial exposure was overwhelming; adaptation followed but emotional impact remained.
**Researchers**	Anticipate that not all gestures will be interpreted as supportive; practice culturally and contextually sensitive responses.	A simple act (offering tissues) was perceived differently by one participant, illustrating that well-intended gestures can trigger unintended reactions.
**Project Managers**	Provide training on managing expectations and maintaining professional boundaries as well regular debriefing	Participants often expected advocacy or material assistance, creating ethical strain.
**Ethics Committees**	Require researcher wellbeing plans alongside participant protection protocols.	Trauma exposure affects both participants and researchers
**Researchers**	Engage in ongoing reflexive practice and monitor signs of moral injury and burnout	Emotional strain and helplessness are foreseeable risks in trauma research.

## Conclusion

Research with traumatized migrant populations demands more than methodological competence; it requires psychological preparedness, ethical clarity, and institutional support. Exposure to traumatic narratives, unmet expectations, and emotionally complex encounters can profoundly affect researchers. Recognizing these risks and embedding structured support mechanisms is essential to safeguarding both researchers and participants in trauma-focused studies.
